# A review of accessibility of administrative healthcare databases in the Asia-Pacific region

**DOI:** 10.3402/jmahp.v3.28076

**Published:** 2015-07-20

**Authors:** Dominique Milea, Soraya Azmi, Praveen Reginald, Patrice Verpillat, Clement Francois

**Affiliations:** 1 Lundbeck Singapore Pte Ltd, Singapore; 2 Azmi Burhani Consulting, Petaling Jaya, Malaysia; 3 Lundbeck SAS, Paris, France; 4 Lundbeck LLC, Deerfield, USA

**Keywords:** administrative, data, database, research, Asia-Pacific, epidemiology

## Abstract

**Objective:**

We describe and compare the availability and accessibility of administrative healthcare databases (AHDB) in several Asia-Pacific countries: Australia, Japan, South Korea, Taiwan, Singapore, China, Thailand, and Malaysia.

**Methods:**

The study included hospital records, reimbursement databases, prescription databases, and data linkages. Databases were first identified through PubMed, Google Scholar, and the ISPOR database register. Database custodians were contacted. Six criteria were used to assess the databases and provided the basis for a tool to categorise databases into seven levels ranging from least accessible (Level 1) to most accessible (Level 7). We also categorised overall data accessibility for each country as high, medium, or low based on accessibility of databases as well as the number of academic articles published using the databases.

**Results:**

Fifty-four administrative databases were identified. Only a limited number of databases allowed access to raw data and were at Level 7 [Medical Data Vision EBM Provider, Japan Medical Data Centre (JMDC) Claims database and Nihon-Chouzai Pharmacy Claims database in Japan, and Medicare, Pharmaceutical Benefits Scheme (PBS), Centre for Health Record Linkage (CHeReL), HealthLinQ, Victorian Data Linkages (VDL), SA-NT DataLink in Australia]. At Levels 3–6 were several databases from Japan [Hamamatsu Medical University Database, Medi-Trend, Nihon University School of Medicine Clinical Data Warehouse (NUSM)], Australia [Western Australia Data Linkage (WADL)], Taiwan [National Health Insurance Research Database (NHIRD)], South Korea [Health Insurance Review and Assessment Service (HIRA)], and Malaysia [United Nations University (UNU)-Casemix]. Countries were categorised as having a high level of data accessibility (Australia, Taiwan, and Japan), medium level of accessibility (South Korea), or a low level of accessibility (Thailand, China, Malaysia, and Singapore). In some countries, data may be available but accessibility was restricted based on requirements by data custodians.

**Conclusions:**

Compared with previous research, this study describes the landscape of databases in the selected countries with more granularity using an assessment tool developed for this purpose. A high number of databases were identified but most had restricted access, preventing their potential use to support research. We hope that this study helps to improve the understanding of the AHDB landscape, increase data sharing and database research in Asia-Pacific countries.

Administrative healthcare databases (AHDB) have become important resources for research in addition to their function of providing the administrative support for which they were first developed (1–4). In many countries, particularly in the US and Europe, AHDB have been used to conduct epidemiological research, pharmacoepidemiology or other types of observational research for several decades (5–13). Databases such as the Veterans Affairs database, or the privately held MarketScan database and the GE Healthcare's Electronic Medical Records database in the United States, as well as the Clinical Practice Research Datalink in the United Kingdom and the PHARMO Database Network database in the Netherlands are some examples of AHDB widely used in research (14–18). This approach has also been taken in studies performed in some countries in Asia, for example, in Taiwan, Korea, and Japan, but not to the extent that it is used in the West (19).

The advantages of utilising AHDB for research are that it allows the use of an existing data set usually containing a population sample that is much larger than what can be incorporated into typical primary data collection studies. It can therefore provide interesting and complementary information to randomised controlled trials about prescribing patterns including off-label use, or patient characteristics without the limitations usually observed when developing prospective studies. As with any secondary data, it often allows researchers to conduct studies in less time compared to primary research since data have already been collected (20). On the other hand, there are limitations to this research method. For example, data are not collected for the sole purpose of any particular study, hence they may not fit the ‘requirements of a specific research question’. Related to this, there is a lack of control over the data collected and data quality may not be as robust compared with primary data collection. There could also be challenges related to the need to rely on proxies for some outcomes of interest, accuracy in data definitions due to changes in administrative procedures or data protection, and privacy issues that must be considered (16).

The use of databases in Western countries has been well documented, and a few earlier studies have compared databases in the US and Europe. One such study by Furu et al. investigated databases in five Nordic countries to assess the possibility of establishing a cross-national data linkage (7). Another study by Groene et al. focussed on the completeness of data in the databases of 177 hospitals in seven European countries and their capacity to allow a multinational hospital performance evaluation (21). Other studies were more interested in disease-specific information from the databases included in their selection. A study by de Groot et al. (22) investigated the use of databases for the purpose of assessing antiepileptic treatment patterns in seven European countries, and a study by Weaver et al. (23) in the United States compared data sources in an evaluation of vaccinations for military veterans.

On the other hand, we were only able to find two articles that discussed the use of AHDBs in Asia. One article described the Asian Pharmacoepidemiology Network, a multinational collaboration, formed for the purpose of supporting and conducting research in the field of pharmacoepidemiology and pharmacovigilance research, including Japan, South Korea, Taiwan, Hong Kong, China, Singapore and joined by US, Australia, and Sweden. This collaborative network involved several AHDB in the Asian region (24). The second article, by Aljunid et al., described the situation related to healthcare data in South Korea, Japan, China, Taiwan, Thailand, and Malaysia (19). This article provided some overall discussion about each government's approach and policies regarding data sharing but did not provide specific details on data access. Overall, the article concluded that accessibility to healthcare data was limited in most of the countries mentioned but did not provide granularity in comparing available information and accessibility to data.

One reason is that AHDB in Asian countries are either inaccessible or accessible to a very limited degree (19). However, this contention has not been well documented or explored in published academic articles. Before setting out on this study, we believed that there could be situations where administrative data may be available for research in countries in the region, which are not clearly described in the public sphere and hence researchers may be simply unaware of their existence. Or in other cases, information on how to access the databases is lacking.

Therefore, we set out to describe AHDBs in various countries in the Asia-Pacific region and to assess database accessibility in Taiwan, South Korea, Japan, China, Thailand, Malaysia, and Singapore. We note that there are also other forms of research databases and data sets that are available, such as large national surveys, research-related data sets, and registries. However, the population sizes included are usually smaller, and the methods of data collection are different to AHDB. Hence, these non-administrative data sets are not included in this study, since the method and purpose of data collection are different.

## Methodology

We selected the countries for this study, based on their upper-middle to high-income status within the Asia-Pacific region, namely Australia, Japan, South Korea, Taiwan, Singapore, China, Thailand, and Malaysia (25). We defined AHDB as databases that collect information primarily for processing and documentation purposes such as registration, transaction, or record keeping for the delivery of a service (26, 27). These include insurance claims databases, prescription databases, and electronic medical records (EMR), as well as data linkage systems that utilise the above. Databases maintained for research purposes such as national health surveys and registries were excluded.

Between June 2013 and January 2014, we used three parallel processes based on freely available Internet resources to find potential databases. First, a PubMed literature search was conducted to find published articles that had used an AHDB to conduct studies. Second, Google Scholar was used to conduct a search of potential administrative databases that provide information on the Internet including national social health insurance agencies. Third, we utilised the ISPOR International Digest of Databases and the non-subscription section of B.R.I.D.G.E. to Data^®^ to corroborate what had already been described by other researchers (28, 29). Information from the three sources was collated, and a further Internet search was performed to obtain details about each database once it was identified. Finally, email and telephone contact details with the database custodians were initiated to obtain additional information such as the process for data access. Outreach through telephone contact was performed in the English language. Databases were categorised into the following types: 1) reimbursement – containing data from insurance claims, 2) prescription – containing data on pharmacy prescriptions, 3) EMR – containing information from medical charts, 4) hospital administration – containing administrative information, and 5) data linkage – containing a combination of various data sources.

The following information was extracted from the literature search: type of administrative databases available by country and type of information available, for example, demographic, prescription, hospitalisation, and cost data. We also noted whether data access was possible for private or public research and what procedures needed to be fulfilled. At a country level, we recorded the information on the administrative databases available and made note of the existence of social or private insurance systems. Finally, we searched for published articles that used the individual databases using PubMed. Our search was non-exhaustive and was meant to give an indication of the frequency of publications rather than a systematic analysis of publication rates.

Next, based on the information we gathered, we defined criteria to categorise the level of data accessibility. These criteria were defined using a simple pragmatic approach by considering potential barriers to data access. Criteria were: 1) Was data access for research allowed? 2) Were contact details for the database custodian publically available? 3) Were there any restrictions to the study question or use by private researchers or industry? 4) Could raw data be provided or must the data custodian conduct the analysis? 5) Must a research team member be a local citizen? 6) Is special collaboration with the database owner required? These criteria allowed the definition of seven levels of data accessibility. The databases were categorised into seven levels of accessibility from least accessible to most accessible, as shown in Table 1. At Level 1, no access and research (whether public or private) is formally allowed. At Level 2, access is unclear since there is lack of information about the database in the public sphere. No direct contact with the data custodian could be established. At Level 3, only limited research is permitted such as research for public interest. At Level 4, raw data are not provided to external researchers and only the results could be made available. At Level 5, there is a requirement that at least one researcher must be a local citizen. At Level 6, collaboration with the data owner is required. Finally, at Level 7, a fee may be levied but none of the above restrictions apply. The levels help to describe pragmatic access and are not intended as a full grading system as some criteria are not mutually exclusive.

**Table 1:** Levels of research access in Asia-Pacific countries - ‘Seven Levels of Data Heaven’

Level	Allows research and information available	No restriction on research question/open to privates	Provision of raw data	Citizenship of researchers involved	Collaboration with data custodian	No technical restrictions	Examples of database
**7**	Access procedure made clear in public sphere	No restriction on research question/open to privates BUT no raw data provided	Raw data provided BUT need a country citizen as part of study	Any citizenship BUT need collaboration with data custodian	Collaboration not required	Fee may be required	Australia: Medicare, PBS, CHeReL, HealthLinQ, VDL, SA-NT DataLink Japan: MDV, JMDC, Nihon-Chouzai
**6**					Any citizenship/collaboration required		Japan: Hamamatsu
**5**				Local citizen required			Australia: WADL Taiwan: NHIRD
**4**			No raw data; data custodian provides results				Japan: Medi-Trend, NUSM Malaysia: UNU-Casemix
**3**		Limited research/no privates/public interest questions only					Japan: DPC South Korea: HIRA
**2**	Access unclear; no direct contact with data custodian could be established						Australia: DVA China: Zhongnan, West China, Jinhua, CHIRA, Zhongshan, URBMI (Guangzhou), UEBMI (Guangzhou), UEBMI (Hebei), UEBMI (Tianjin), Minhang, GPH, PLA, PLAGH, Soochow, NRCMS Japan: CGRN, Osaka, COS, Kyoto, NHI, JMIRI, LTCI Malaysia: EHMIS Singapore: NEHR South Korea: NHIC Thailand: UCS, SSS, CSMBS, Ramathibodi, Buddhachinaraj, Sunpasitthiprasong, Nakhon Thai
**1**	No access						Japan: JammNet Singapore: Medisave

Medicare=Medicare Australia; PBS=Pharmaceutical Benefits Scheme; CHeReL=Centre for Health Record Linkage; VDL=Victorian Data Linkages; MDV=Medical Data Vision EBM Provider; JMDC=Japan Medical Data Centre Claims database; Nihon-Chouzai=Nihon-Chouzai Pharmacy Claims database; Hamamatsu=Hamamatsu Medical University database; WADL=Western Australia Data Linkage; NHIRD=National Health Insurance Research Database; NUSM=Nihon University School of Medicine Clinical Data Warehouse; UNU=United Nations University; DPC=Diagnosis Procedure Combination Database; HIRA=Health Insurance Review and Assessment database; DVA=Department of Veterans Affairs Claims database; Zhongnan=Zhongnan Hospital, China; West China=West China Hospital Electronic Medical Record, China; Jinhua=Jinhua Municipal Central Hospital database, China; CHIRA=China Health Insurance Research Association database, China; Zhongshan=Zhongshan Hospital Electronic Medical Record, China; URBMI (Guangzhou)=Urban Resident Basic Medical Insurance database, Guangzhou, China; UEBMI (Guangzhou)=Urban Employee Basic Medical Insurance database, Guangzhou, China; UEBMI (Hebei)=Urban Employee Basic Medical Insurance database, Hebei, China; UEBMI (Tianjin)=Urban Employee Basic Medical Insurance database, Tianjin, China; Minhang=Electronic Health Records (EHR) system – Minhang, Shanghai, China; GPH=Guangzhou Psychiatric Hospital database, China; PLA=The 306th Hospital of P.L.A Beijing, China; PLAGH=Chinese People's Liberation Army General Hospital database; Soochow=Soochow University Affiliates Children's Hospital database; NRCMS=New Rural Cooperative Medical Scheme database; CGRN=Convergence CT Global Research Network, Japan; Osaka=Osaka University Database, Japan; COS=Computer Ordering System Database Dokkyo Medical University, Japan; Kyoto=Electronic Medical Record Retrieval System-Kyoto University Hospital; NHI=National Health Insurance Database; JMIRI=JMIRI Pharmacy Claims Database; LTCI=Long-Term Care Insurance; EHMIS=Electronic Health Management Information System; NEHR=National Electronics Health Records Database; NHIC=National Health Insurance Corporation database, South Korea; UCS=Universal Coverage Scheme Database; SSS=Social Security Scheme Database; CSMBS=Civil Servants’ Medical Benefit Scheme Database; Ramathibodi=Ramathibodi Hospital Electronic Medical Record; Buddhachinaraj=Buddhachinaraj Hospital database; Sunpasitthiprasong=Sunpasitthiprasong Hospital Electronic Medical Record; Nakhon Thai=Nakhon Thai Crown Prince Hospital Electronic Medical Record; JammNet=JammNet database, Japan; Medisave=Medisave database, Singapore.

Finally, countries were divided into categories of high, medium, or low accessibility, depending upon the availability of administrative databases as well as the ease in accessing the data. We also took into account the number of publications published using country-level databases as an indication of accessibility to data, although it also reflects awareness and interest on this type of data source for research. Countries with high data accessibility were countries that had administrative databases with large population coverage that were also accessible to researchers and for which application processes were plainly described. Countries were described as having medium data accessibility when access to databases was allowed, but had conditions or limitations. Countries were described as having low data accessibility when database coverage was limited, information was not available, or databases did not allow access.

## Results

### Levels of data accessibility

We found 54 administrative databases in the eight selected countries. Individual databases in the same country could have different levels of accessibility depending on the ease of fulfilling access requirements (Table 1). Based on the seven levels of accessibility, examples of databases in the highest level of accessibility, Level 7, were the Medicare, Pharmaceutical Benefits Scheme (PBS), Centre for Health Record Linkage (CHeReL), HealthLinQ, Victorian Data Linkages (VDL), and SA-NT DataLink in Australia; and the Medical Data Vision EBM Provider, Japan Medical Data Centre (JMDC) Claims database, and Nihon-Chouzai Pharmacy Claim database in Japan. Examples of databases at Level 6 were the Hamamatsu Medical University Database in Japan. Examples at Level 5 were the National Health Insurance Research Database (NHIRD) in Taiwan and the Western Australia Data Linkage (WADL) in Australia. At Level 4 were the Medi-Trend database and Nihon University School of Medicine (NUSM) Clinical Data Warehouse in Japan, and the United Nations University-Casemix (UNU-Casemix) database in Malaysia. At Level 3 were Health Insurance Review and Assessment Service (HIRA) database in South Korea and the Diagnosis Procedure Combination (DPC) database in Japan. At Level 2 were all databases in Thailand and China, and the Electronic Health Management Information System (EHMIS) in Malaysia. Finally, at Level 1, were the Medisave database in Singapore and the JammNet database in Japan.

### Accessibility of databases by country

Table 2 provides a qualitative assessment of data accessibility by country. It also describes the type of database, number of publications, and type of information available in the database. Countries with high data accessibility were Taiwan, Australia, and Japan; medium data accessibility was South Korea; low data accessibility were Thailand, China, Malaysia, and Singapore.

**Table 2:** A list of administrative databases by country, type of administrative database, accessibility, and available information

						Type of information available			
						
Countries	Accessibility of data for research	Availability of social insurance	Administrative databases	Type of administrative database	Number of publications	Demographic data	Prescription data	Hospitalisation data	Cost data
**Taiwan**	High	Yes	NHIRD	Reimbursement	+++	√	√	√	√

**Australia**	High	Yes	Medicare Database	Reimbursement	++	–	–	–	√
			Pharmaceutical Benefit Scheme (PBS) Database	Prescription	++	–	√	–	√
			Western Australia Data Linkage	Data linkage	+	√^a^	√^a^	√^a^	√^a^
			Centre for Health Record Linkage (CHeReL)	Data linkage	+	√^a^	√^a^	√^a^	–
			HealthLinQ	Data linkage	+	√^a^	√^a^	√^a^	–
			Victorian Data Linkages	Data linkage	+	√^a^	√^a^	√^a^	√^a^
			SA-NT Datalink	Data linkage	+	√^a^	–	–	–
			Department of Veterans Affairs Claims Database	Reimbursement	+	√^a^	√^a^	√^a^	–

**Japan**	High	Yes	Convergence CT Global Research Network (CGRN)	EMR	+	√	√	√	–
			Medical Data Vision (MDV) EBM Provider	Hospital administration	+	√	√	√	√
			Hamamatsu Medical University Database	EMR	+	√	√	√	–
			Osaka University Database	EMR	++	√	√	√	–
			Computer Operating System Database Dokkyo Medical University	EMR	+	√	√	√	–
			EMR Retrieval System-Kyoto University Hospital	EMR	+	√	√	–	–
			Nihon University School of Medicine Clinical Data Warehouse	EMR	+	√	√	√	√
			Japan Medical Data Centre (JMDC) Claims database	Reimbursement	+	√	√	√	√
			JammNet	Reimbursement	+	√	√	√	–
			Diagnosis Procedure Code Database	EMR	++	√	–	√	√
			NHI Database	Reimbursement	+	√	√	√	√
			Medi-Trend	Prescription	+	√	√	–	√
			Nihon-Chouzai Pharmacy Claim Database	Prescription	+	√	√	–	√
			JMIRI Pharmacy Claims Database	Prescription	+	√	√	–	-
			Long term care insurance (Kaigo Hoken)	Reimbursement	+	√	√	√	√

**South Korea**	Medium	Yes	NHIC database	Reimbursement	++	√	√	–	√
			HIRA database	Reimbursement	++	–	√	√	√

**Thailand**	Low	Yes	Universal Coverage Scheme	Reimbursement	+	√^b^	√^b^	√^b^	√^b^
			Civil Servant Medical Benefits Scheme	Reimbursement	+	√^b^	√^b^	√^b^	√^b^
			Social Security Scheme	Reimbursement	+	√^b^	√^b^	√^b^	√^b^
			Ramathibodi Hospital Database	EMR	+	√^b^	√^b^	√^b^	√^b^
			Buddhachinaraj Hospital Database	EMR	+	√^b^	√^b^	√^b^	√^b^
			Sunpasitthiprasong Hospital	EMR	+	√^b^	√^b^	√^b^	√^b^
			Nakhon Thai Crown Prince Hospital	EMR	+	√^b^	√^b^	√^b^	√^b^

**China**	Low	Yes	Zhongnan Hospital	EMR	+	√^b^	√^b^	√^b^	√^b^
			West China Hospital	EMR	+	√^b^	√^b^	√^b^	√^b^
			Jinhua Municipal Central Hospital database	EMR	+	√^b^	√^b^	√^b^	√^b^
			The 306th Hospital of PLA, Beijing	EMR	+	√^b^	√^b^	√^b^	√^b^
			Chinese People's liberation army general hospital database	EMR	+	√^b^	√^b^	√^b^	√^b^
			Soochow university affiliates children's hospital database	EMR	+	√^b^	√^b^	√^b^	√^b^
			UEBMI (Tianjin)	Reimbursement	+	√^b^	√^b^	√^b^	√^b^
			UEBMI (Guangzhou)	Reimbursement	+	√^b^	√^b^	√^b^	√^b^
			UEBMI (Hebei)	Reimbursement	+	√^b^	√^b^	√^b^	√^b^

**China** ***(cont'd)***	Low	Yes	URBMI (Guangzhou)	Reimbursement	+	√^b^	√^b^	√^b^	√^b^
			CHIRA database	Reimbursement	+	√^b^	–	√^b^	√^b^
			Zhongshan Hospital	EMR	+	√^b^	–	–	√^b^
			New Rural Cooperative Medical Scheme (NRCMS)	Reimbursement	+	√^b^	√^b^	√^b^	√^b^
			Electronic Health Records (EHR) system – Minhang, Shanghai	EMR	+	√^b^	–	√^b^	√^b^
			Guangzhao Psychiatric Hospital	EMR	+	√^b^	–	√^b^	–

**Singapore**	Low	Yes	Medisave database	Reimbursement	+	√	√^b^	√^b^	√^b^
			National Electronics Health Records Database	Hospital database	+	√	√	√	–

**Malaysia**	Low	No	UNU-Casemix database	Hospital administration (discharge records)	+	√	–	√	√
			Electronic Health Management Information System	Hospital administration	+	√	–	√	–

a The data are available through various databases linked by the data linkage system and approval by the relevant data custodians need to be sought.

b The availability of such data is based on assumption and from previous studies on the database.

+ Less than 10 publications ++ Between 10 and 100 publications +++ More than 100 publications

#### High data accessibility

In countries categorised as having high data accessibility, a large amount of data is held within the administrative databases, and contact information was found through the Internet. Overall, there were many published articles from these countries. In most cases, a more detailed level of information of database variables was available on the database website – for example, information from the NHIRD (30) in Taiwan, the PBS (31, 32), and Medicare Australia (31, 32) in Australia. In Taiwan, students and researchers may use a random sample data set consisting of 1 million patients or make study-specific requests for data (33). In Australia, accessible databases include the PBS database, which contains prescription data of medications listed in the PBS schedule, while Medicare Australia contains social insurance data. There are also other databases available for research. In Japan, several universities have accessible databases and there are also privately held databases, such as the JMDC Claims database and the Medical Data Vision EBM Provider database that can also be accessed.

#### Medium data accessibility

South Korea was categorised as having medium data accessibility. South Korea has the HIRA database and the National Health Insurance Corporation (NHIC), which are linked to the National Health Insurance programme. Both databases have been widely used for local studies in South Korea (34–42). According to an email reply from HIRA, the database is only available for academic research of public interest and is not accessible to international researchers, private individuals, or organisations. After an attempt to email as well as call, we remained unable to reach the appropriate person representing the data custodians of the NHIC database.

#### Low data accessibility

China, Thailand, Singapore, and Malaysia were categorised as having low data accessibility. Although all four countries have large administrative databases, access to the data is more challenging, either because of more restrictive requirements or due to a lack of information about the appropriate contact point.

In Thailand, available databases were the Universal Coverage Scheme (UCS), Social Security Scheme (SSS), and Civil Servants’ Medical Benefit Scheme (CSMBS). Several hospital EMR systems have also been used in Thailand for research purposes as described in Table 2. Contact details for the database owners could not be obtained, but research is probably possible as shown by published studies by academics who have used the databases (43–49). China has social insurance databases, which include the Urban Resident Basic Medical Insurance (URBMI), Urban Employee Basic Medical Insurance (UEBMI), and the New Rural Cooperative Medical Scheme (NRCMS). Several studies have utilised these databases at a provincial level and findings have been presented as conference posters, showing that access is possible (50, 51). There were also several hospital EMR systems that have been used in past research studies (Table 2). However, contact information of the database custodians was not available.

Singapore and Malaysia were categorised as having low data accessibility for several reasons. In both cases, there was a lack of publicly available information about administrative databases, and there were few studies utilising the databases in these countries (52–56). For Medisave Singapore, an email reply from the database custodians stated that external research was not allowed. The Malaysian government has established an EHMIS for its public hospitals, but the systems may still be in the process of implementation (19, 57). Currently, no information is available in the public sphere that indicates a point of contact for access to the database. On a smaller scale, the United Nations University (UNU)-Casemix database includes data from two academic centres. Limitations of this database include the lack of prescription data within the database, and that raw data are not accessible to researchers. Researchers interested in obtaining data for research would be required to work with the database custodian to obtain the statistical analysis of the data.

## Discussion

Our study was intended to provide granularity and greater depth of understanding on the landscape of AHDBs in Asian countries. When compared to Western countries, it would seem that there are only a few countries in Asia that can be considered as having AHDB which are widely accessible for research. These include Australia, Japan, and Taiwan, which are countries with more advanced medical research environments and are among the more economically advanced countries in the region. Although South Korea has a well-developed database, accessibility to data had some limitations at the time of our study. For example, access to data was said to be limited to academic researchers and research of public interest.

However, as can be expected, some databases in the same country may be more accessible than others. For instance in Malaysia, the EHMIS is not available for research but the UNU-Casemix system database could potentially be used for research. Similarly, some databases in Japan are more easily accessible (such as the JMDC or MDV) compared to others like JammNet. Hence, it would not be appropriate to describe all databases in one country or the region as being inaccessible. This differentiation is more apparent when we consider the different levels of data accessibility that we have described. The levels of accessibility are intended as a tool for the purpose of understanding of databases in these countries to assist researchers and not to judge the databases or countries. In our suggested categorisation, databases at Level 7 are considered fully accessible and open to researchers with minimal challenges, while databases at Level 1 are not accessible. Depending on the database, there were different requirements for researchers – for instance, whether limited to national citizens or a lack of information about access procedures. However, depending on the requirements, it might be possible to overcome some of these challenges. Using this more granular approach to describe accessibility could help promote understanding among researchers, as well as possibly to develop more collaborative discussions with data custodians.

We categorised the AHDBs into seven levels of accessibility that can be regarded as ‘Seven Levels of Data Heaven’. The concept of a data paradise has previously been mentioned in a report for the U.S. National Academy of Science discussing the importance of clinical data as a public good. The report related such a ‘paradise’ as when data are ‘recognised as a staple that should be widely available and integrated across sites and practices’ (58). Yet, it is understandable that there may be several reasons for the lower levels of accessibility. First, the concept of database research is fairly new in most countries in Asia. In the example of the Malaysian EHMIS, it is possible that the lack of data sharing may not be due to procedural limitations but to a lack of readiness at the current time. Discussions at the 2014 ISPOR Asia-Pacific conference in Beijing indicated that this may also be the case in countries like China. Related to this, other issues may arise, such as the lack of process definition or custodians still being wary of the risks and uncertain of the benefits that might be gained. Due to the novelty of the concept of data sharing and transparency, there could be also a lack of trust by data custodians that database research could be conducted ethically while still protecting patient privacy and confidentiality. Yet, in many developed nations, database research has been used to strengthen knowledge and research. Based on the experience of countries like Taiwan, such processes can be instituted to protect the rights of patients and citizens while still ensuring that data can be used for medical research and public health purposes. Among these processes are ethics approvals and de-identification of personal data. Finally, the publication of medical evidence found through this type of research also helps to keep the use of administrative data transparent.

Our study is not without limitations. Firstly, the study assessment was conducted in a qualitative manner particularly with regard to measuring accessibility of the databases. Secondly, there were only eight countries included in the study, and the study was also limited to administrative databases. Thirdly, telephone calls to the data custodian were performed in English, thus the level of understanding we could achieve was also dependent on the English-language usage at the organisation we contacted. If we had used the language of the country, information gained may have been slightly different. This is reflected in cases where there are publications in the past, but we were not able to obtain a response during our outreach. Fourthly, we did not utilise the subscribed portion of B.R.I.D.G.E. to Data^®^, a private database listing containing information on databases internationally. Other data sources such as disease registries and national surveys were not included, even if such sources also hold potential for secondary research and are available in some countries. Access procedures to databases clearly can change with time. After our research was performed, there was a recent change at HIRA that now allows researchers to access a sample data set (59). Our ‘Accessibility Levels’ did not take into account the fee required for data access. In some cases, fees for data access could be high. It would have been difficult to set objective criteria for high level fees, since the fee would be dependent on study scope, and furthermore the high cost of using US commercial data has not prevented many studies being conducted. The delineation of the levels of access is also imperfect and can be debatable; some databases may have overlapping requirements from other criteria.

It is foreseeable that countries in Asia that recognise the administrative function of databases and also allow research could be at an advantage compared to those that do not. The availability of large data sets can create synergies between research and innovation, since it enables the study of disease and treatment patterns among patients. It is possible to do this even while ensuring that data privacy and patient confidentiality are in place. For many developing countries in Asia, data sharing could help build a strong research environment that would benefit the country through better understanding of disease patterns within its population. Local published research strengthens evidence-based medicine and can assist in healthcare policy decisions in a more directly relevant way. Aside from benefits to public health and medicine, this can also help spur innovation and a knowledge-based economy. As was recently the case in South Korea, it is hoped that more countries will move up to the higher levels of database access in the future, as this would help to increase understanding of disease patterns in Asia.

## Conclusion

To the best of our knowledge, there has been no published study that has described and compared the information availability and accessibility focussing on administrative databases in Asia-Pacific countries. We sought to bridge the gap in information and provide greater granularity and promote understanding of the landscape of administrative databases in the region.

Unlike the Western hemisphere, administrative databases are less widely accessible in Asia, but we have been able to identify some databases that allow research with less challenging requirements. Our study provides insight into the different levels of access to data in eight Asian countries. Levels of access differ according to country and individual databases. We suggest a tool – Seven Levels of Data Heaven – to differentiate accessibility of databases, which we hope can help to increase understanding of databases in the Asian countries that we studied. We have also listed the levels of accessibility of several databases in different countries to provide greater granularity of information. We hope that a greater understanding of the database landscape can help to identify progressive steps to increase data sharing and data usage for research purposes even if it means to increase access by removing one requirement. Finally, we hope that this would lead to improved understanding of disease patterns and the management of healthcare conditions for the betterment of patients in this region.
